# Scalp attached tangential magnetoencephalography using tunnel magneto-resistive sensors

**DOI:** 10.1038/s41598-022-10155-6

**Published:** 2022-04-12

**Authors:** Akitake Kanno, Nobukazu Nakasato, Mikihiko Oogane, Kosuke Fujiwara, Takafumi Nakano, Tadashi Arimoto, Hitoshi Matsuzaki, Yasuo Ando

**Affiliations:** 1 Department of Epileptology, Tohoku University Graduate School of Medicine, Sendai, Miyagi Japan; 2 Department of Advanced Spintronics Medical Engineering, Graduate School of Engineering, Tohoku University, Sendai, Miyagi Japan; 3 Department of Applied Physics, Graduate School of Engineering, Tohoku University, Sendai, Miyagi Japan; 4 Spin Sensing Factory Corp., Sendai, Miyagi Japan; 5 KONICA MINOLTA, INC., Tokyo, Japan

**Keywords:** Somatosensory system, Applied physics, Neuroscience, Biomedical engineering

## Abstract

Non-invasive human brain functional imaging with millisecond resolution can be achieved only with magnetoencephalography (MEG) and electroencephalography (EEG). MEG has better spatial resolution than EEG because signal distortion due to inhomogeneous head conductivity is negligible in MEG but serious in EEG. However, this advantage has been practically limited by the necessary setback distances between the sensors and scalp, because the Dewar vessel containing liquid helium for superconducting quantum interference devices (SQUIDs) requires a thick vacuum wall. Latest developments of high critical temperature (high-*T*_*c*_) SQUIDs or optically pumped magnetometers have allowed closer placement of MEG sensors to the scalp. Here we introduce the use of tunnel magneto-resistive (TMR) sensors for scalp-attached MEG. Improvement of TMR sensitivity with magnetic flux concentrators enabled scalp-tangential MEG at 2.6 mm above the scalp, to target the largest signal component produced by the neural current below. In a healthy subject, our single-channel TMR-MEG system clearly demonstrated the N20m, the initial cortical component of the somatosensory evoked response after median nerve stimulation. Multisite measurement confirmed a spatially and temporally steep peak of N20m, immediately above the source at a latency around 20 ms, indicating a new approach to non-invasive functional brain imaging with millimeter and millisecond resolutions.

## Introduction

Electroencephalography (EEG) and magnetoencephalography (MEG) are the only methods of non-invasive imaging of human brain function that can achieve millisecond time resolution by electrical or magnetic neural current measurement^1^. MEG has theoretically higher spatial resolution than EEG because MEG suffers negligible signal distortion due to inhomogeneous head conductivity, whereas EEG shows serious distortion^1,2^. However, the spatial resolution of MEG has been practically limited by the necessary setback distance between the sensors and scalp. The development of MEG sensors attached to the scalp has long been a target of research because magnetic field strength attenuates according to the square root law of sensor-source distance^3^.

Conventional MEG sensors, called superconducting quantum interference devices (SQUIDs), require ultra-low operating temperatures (− 269 °C) only achieved by liquid helium, so a Dewar vessel with thick vacuum walls is required to contain the liquid helium and the sensors^1,2^. Another problem is the construction of the helmet-shaped Dewar vessel to cover the entire head. The fixed shape greatly increases the setback distance, in order to accommodate the individual variability in head size and shape^1,2^. Latest developments in high-*T*_*c*_ SQUIDs^4,5^ or optically pumped magnetometers (OPMs)^6,7^ have allowed closer MEG measurement to the scalp. However, perfect attachment of the MEG sensors to the scalp has not yet been achieved.

Here we introduce the use of tunnel magneto-resistive (TMR) sensors based on magnetic tunnel junctions (MTJs) that operate at room temperature^8,9^. We have already reported successful measurement of chest-attached magnetocardiography (MCG) without signal averaging techniques and scalp-attached MEG of the alpha wave, awake brain background rhythm, by signal averaging techniques^10^. Our combination of magnetic flux concentrators has finally equaled the sensitivity of the TMR sensor at the level of 940 fT/√Hz at 1 Hz and 50 fT/√Hz at 1 kHz^11^, which are adequate to measure the somatosensory evoked magnetic fields (SEFs) most commonly used in clinical functional mapping of MEG^12–15^. Our present success with scalp-attached tangential MEG measurement will allow non-invasive imaging of human brain functions at millimeter and millisecond resolutions.

## TMR sensors

**Figure 1:**
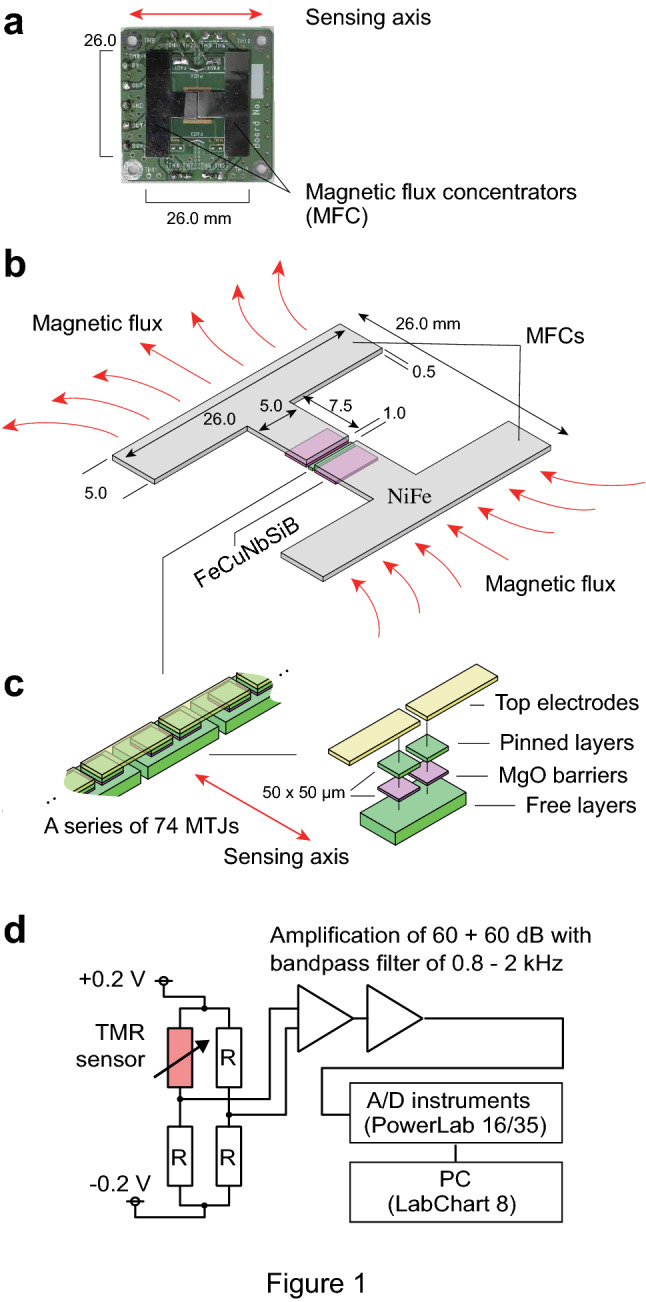
TMR sensor system used for scalp-attached tangential MEG. (**a**) Photograph of a TMR-MEG system consisting of two T-shaped MFCs (black) mounted on a circuit board. (**b**) Schema of two MFCs fabricated by two film deposition of 300 nm-thick FeCuNbSiB in a size of 5.0 mm × 2.5 mm (purple) and a facing pair of T-shaped 0.5 mm-thick NiFe plates with 26.0-mm width (gray) parallel to the array of 74 MTJ (green). (**c**) Partial and magnified schemas of the array of 74 MTJs. Resistance changes, across pinned and free layers (green) separated by thin MgO barriers (purple), represent the scalp surface tangential MEG through MFCs. Size of each free layer in the 74 MTJ arrays is 100 μm × 150 μm × 140 nm. Top electrodes (yellow) connect the MTJs into a bridge circuit, amplifiers, and a signal analysis computer. (**d**) Measurement circuit diagram used for the MEG measurement. A TMR sensor was used as one of the resistors in the bridge circuit, and its output voltage was amplified and filtered. The bridge was supplied with ± 0.2 V, so the bias voltage of about 0.2 V was applied to the TMR sensor. The circuit to receive the signal from the bridge consisted of our homemade amplifiers and capacitor-resistor passive filters. The biomagnetic field signal measured by the TMR sensor was input to a two-stage amplification circuit with a total gain of 120 dB and passed through an analog bandpass filter from 0.8 Hz to 2 kHz. The amplified and filtered signals were recorded on a PC at sampling rate of 10 kHz using an ADInstruments PowerLab 16/35 of 16 bit resolution and LabChart8 software. The magnetic field signals were filtered in the software with a moving average (digital filter) from 20 to 200 Hz.

Figure 1 shows our TMR sensor system for scalp-attached tangential MEG. The core of the TMR sensor consists of MTJs developed based on recent spintronics science^8,9^. At room temperature, the magnetic field can be measured as resistance change through MTJs. Recently, marked improvement in the sensitivity has enabled us to perform MCG even without signal averaging techniques, as well as detect the MEG signal of the alpha rhythm, the background brain activity during wakefulness, with averaging techniques in a healthy subject^10^. We have now dramatically improved the signal-to-noise ratio of the TMR sensor by optimizing the film structure of the MTJs, integrating the MTJ devices, combining a facing pair of T-shaped magnetic flux concentrators (MFCs), and developing a low-noise amplifier circuit. The sensitivity of our new TMR sensor has achieved the level of 940 fT/√Hz at 1 Hz, 200 fT/√Hz at 10 Hz, and 50 fT/√Hz at 1 kHz^11^, which has about 100 times better sensitivity compared to previous magneto-resistive sensors^16^. Our TMR sensor has inferior sensitivity compared to OPM and SQUID sensors at this point, but superior to other highly sensitive magnetic sensor devices, such as magneto-impedance sensors^17^, fluxgate sensors^18^, or diamond quantum sensors^19^. Intrinsic bandwidth of our TMR sensor extends from direct current to about 1 GHz, corresponding to the resonant frequency of the free layer material CoFeSiB^20^.

## Advantages of scalp “tangential” MEG

**Figure 2:**
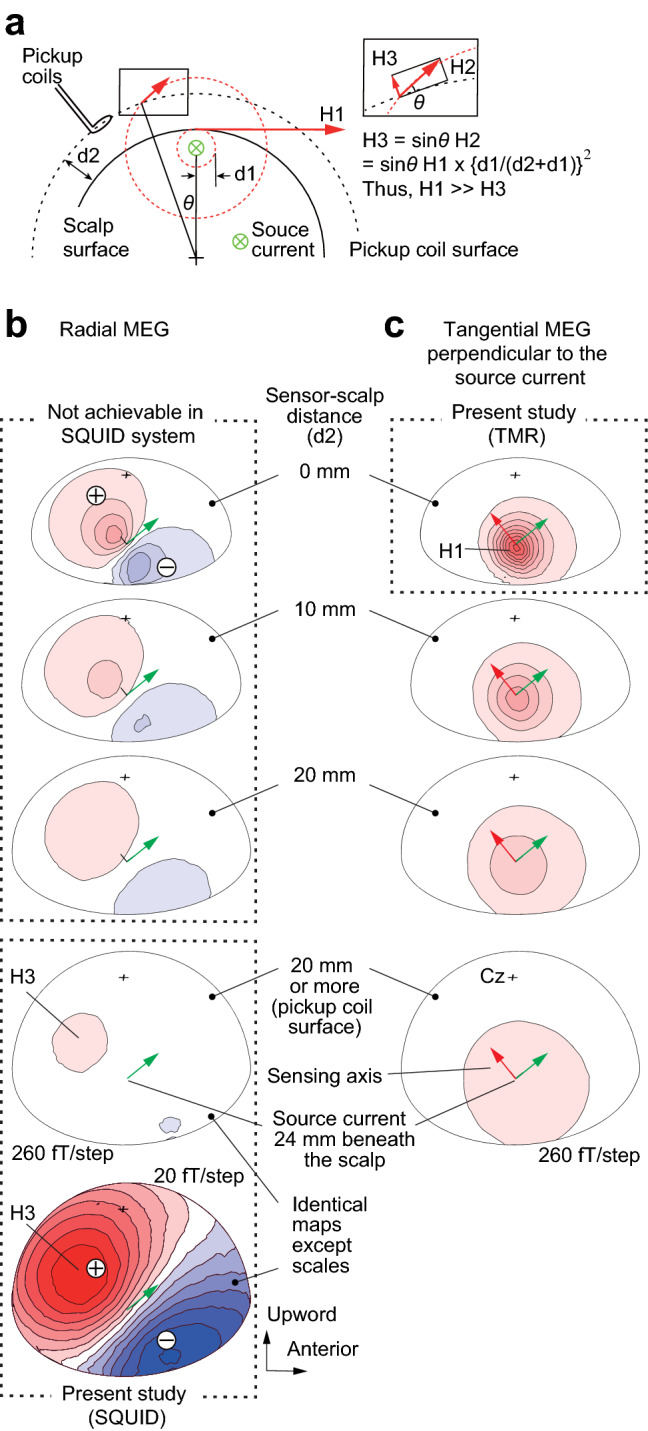
Advantages of scalp-attached tangential MEG. (**a**) Schema to explain the difference in amplitude maxima between scalp-attached tangential MEG measured by TMR sensors (H1), unmeasured oblique MEG (H2), and scalp radial MEG measured at the pickup coil surface of SQUID (H3). Note the H1 amplitude is much larger than H3 under the Bio-Savart law showing the magnetic field generated by a current is negatively correlated to the square of source-sensor distance (d1 for H1 and d1 + d2 for H2). (**b**) Right parietal view of simulated isofield maps of radial MEG at 4 different setback distances between the sensors and the scalp. A current source positioned 24 mm beneath the scalp simulating the N20m response after the left median nerve stimulation demonstrated in the present study. Note the clear dipole patterns of short inter-peak distances between magnetic influx and outflux, measured at 0, 10, and 20 mm above the scalp, are not achievable with superconducting MEG due to the wall thickness of the Dewar vessel containing liquid helium. (**c**) Simulation of tangential MEG maps when measured perpendicularly to the source current. Note the largest and steepest peak on the scalp immediately above the source (H1) proved in the present study.

**Figure 3:**
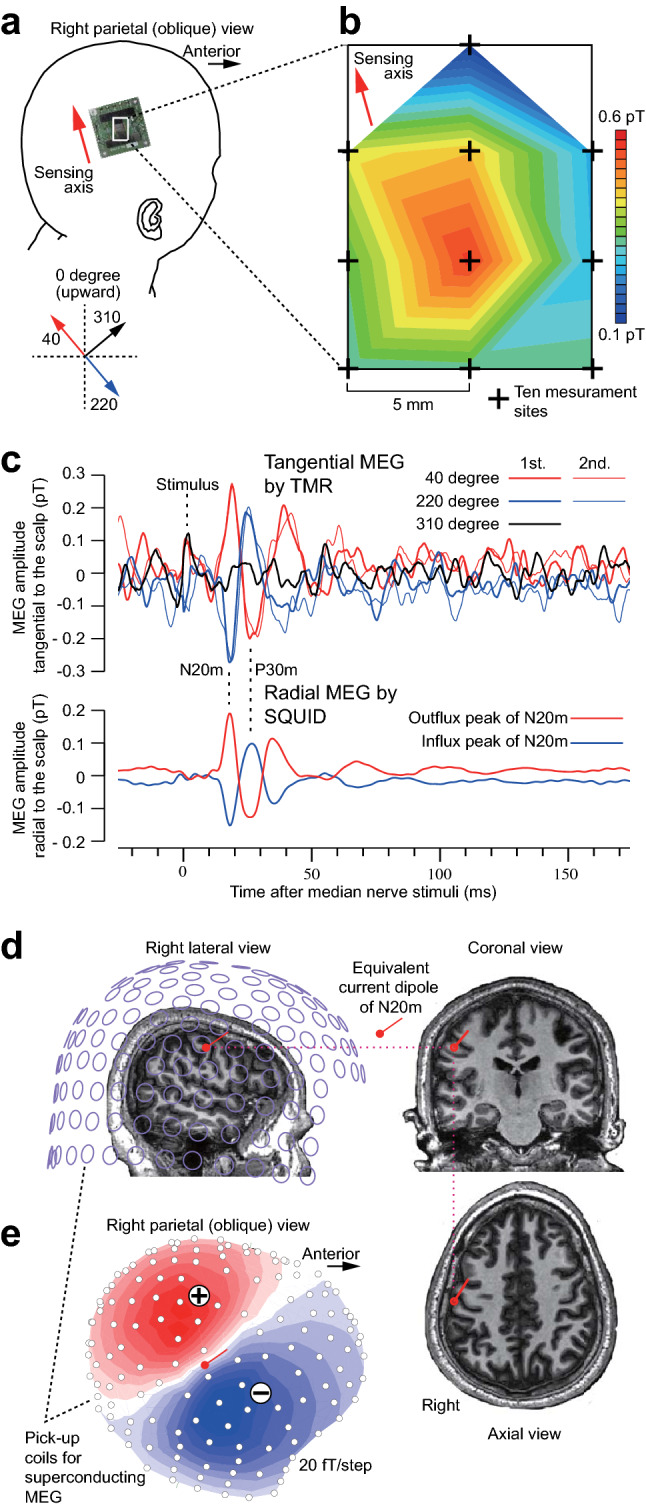
Comparison of tangential MEG using TMR sensors and radial MEG using SQUIDs to measure SEFs in a healthy subject. (**a**) Photograph of a single channel TMR-MEG system placed on the scalp over the right parietal area. Scalp-attached MEG was performed at 2.63 mm from the scalp to the center of the magnetic sensing point. (**b**) Isofield map of N20m, the first SEF component for the left median nerve stimulation, measured at 10 different sites with identical sensing axis of 40 degree counterclockwise from superiorly. Note the spatially steep peak of the N20m. (**c**) Representative SEF waveforms measured by scalp-attached tangential TMR-MEG along three different sensing axes (top) and by the helmet-shaped 200-channel superconducting MEG system (bottom). The 1st and 2nd lines represent two different averaging sessions (N = 9000) to show waveform reproducibility in TMR-MEG for the first (N20m) and second (P30m) components of SEFs comparable to superconducting MEG. (**d**) The helmet-shaped 200-channel radial SQUID-MEG and the equivalent current dipole of N20m superimposed on sagittal, coronal, and axial magnetic resonance (MR) images of the subject. Note the setback distance between the pickup coils and the scalp due to the Dewar vessel containing liquid helium. A red circle and a bar respectively indicate position (on the central sulcus) and orientation (anterior and superior) of the N20m dipole. (**e**) Isofield map of N20m measured by a helmet-shaped 200-channel radial SQUID-MEG. N20m dipole is projected to the coil surface.

Figure 2 compares the radial and tangential measurements of MEG, simulating the conventional SQUID-MEG system and our TMR-MEG system, respectively, for different sensor positions on the scalp. Isofield maps of the N20m, the initial cortical component of SEF after median nerve stimulation, were calculated based on actually measured data using a helmet-shaped MEG system with 200 channel SQUID sensors (Fig. 3c–e). The shorter setback distance between the MEG sensors and the scalp obtained larger signal amplitude and more localized signal distribution in both radial and tangential maps.

In the radial maps simulating conventional SQUID-MEG measurements (Fig. 2b), a single tangential neural current produces the null field at the scalp immediately above the source, but a pair of outflux and influx peaks, called the single dipole pattern, are spatially separated according to the distance function between the sensors and the source^2^. Mathematical models are necessary to estimate the location and orientation of the neural current underneath the scalp^2^. If there are two or more neural sources, separation of these sources becomes difficult in radial MEG because the multiple dipole patterns spatially overlap. In conventional radial MEG systems, the pickup coil surfaces have setback distances of 20 mm or more. Consequently, separation of multiple sources has been challenging in many MEG applications, especially for clinical use, unless only two or a limited number of sources are separated far enough for visual separation^21,22^ or with the use of highly sophisticated mathematical models with certain limitations for nearby sources^23^.

In the tangential maps simulating our new TMR-MEG (Fig. 2c), a single tangential neural current produces the highest signal intensity at the scalp immediately above the source as the sensing axis is set perpendicularly to the source current. Mathematical models may not be necessary to identify the scalp position immediately above the source if multisite measurement (Fig. 3b) is available or a high-density multichannel system could be used in the future.

## Demonstration of scalp surface and scalp tangential TMR-MEG

We measured the SEFs for left median nerve stimuli using a newly developed TMR sensor in a subject (Fig. 3a–c). Based on the N20m source position and orientation previously estimated by SQUID-MEG (Fig. 3d,e), we placed a TMR sensor on the right parietal scalp directly to measure the tangential component of MEG (Fig. 3a). The sensing axis of the sensor was set to measure perpendicular, parallel, and opposite perpendicular components to the N20m current orientation. We confirmed clear and reproducible N20m peaks and the following P30m components of the SEF tangentially to the scalp. These waveforms were almost identical to the radial components of N20m and P30m measurements obtained by SQUID-MEG (Fig. 3c). Then we placed the TMR sensor at 10 different grid sites at 5-mm distances to visualize the isofield map of the tangential N20m over the scalp (Fig. 3b) and found a spatially steep peak of N20m confirming our previous simulation (Fig. 2c).

## Discussion

The present study succeeded in scalp-attached and scalp-tangential measurement of SEFs using a newly developed TMR sensor that operates at room temperature. Multisite measurement using the single channel TMR sensor identified a spatially steep N20m peak immediately above the source, showing higher spatial resolution than conventional SQUID-MEG systems. Our findings indicate the potential of TMR-MEG for non-invasive functional brain imaging with millimeter and millisecond resolutions.

Our findings suggest that the TMR sensor is very promising MEG device to cover the entire head with no setback distance from the scalp. Recently, a liquid nitrogen-cooled cryostat was built to house a 7-channel high-Tc SQUID placed at 1–3 mm from the head surface^4^. However, a helmet-shaped rigid cryostat is necessary in practice to cover the entire head, even for high-Tc SQUIDS, so increasing the setback distance from the scalp to the order of a few centimeters. A flexible (EEG-like) cap for OPM was also designed in which the minimum distance from the scalp to the outer casing of vapor cell was as small as 1.9 mm^24^. Distance from the outer casing to the OPM sensing point is 6.0 mm or more^24–26^, because the vapor cell is electrically heated to around 150 °C to achieve optimum ^87^Rb vapor density^25,26^, and because the vapor cell must be set to reduce the surface temperature of the outer sensor housing^26^. Thus, we assume the total setback distance from the scalp to the OPM sensing point is about 7.9 mm or more^24^. In the present study, the setback distance from the scalp to the TMR sensor was 2.6 mm. We can technically reduce the distance to zero (perfect attachment to the scalp) by placing the sensor board upside down. We can also design a flexible cap for scalp-attached TMR to cover the entire head in the future.

TMR has several known advantages which may allow revolutionary development of MEG for functional human brain imaging and clinical application in addition to scalp-attached measurement^27^. First, the wide dynamic range above 100 μT of our TMR sensors may enable MEG measurement outside the magnetically shielded room. Using our prototype gradiometric TMR sensors with the cancelled environmental noise level of 40 pT/√Hz, we have already succeeded in measuring averaged MCG outside the magnetically shielded room. Second, the fabrication and running costs of TMR sensors are far lower than any other biomagnetic sensors^28^. Third, the MTJs, the core of the TMR sensors, are much smaller than any other biomagnetic sensors. The spatial resolution of MEG depends on the sensor spacing: smaller distance between the sensors provides better discrimination between neural sources^5^. In the present study, the whole sensor package was 26 × 26 mm, corresponding to the MFC size. If the MTJ detectivity can be increased by 10 times, the MFC size can be reduced to 1/10 (2.6 × 2.6 mm). We believe this target can be soon achieved, since the detectivity for our TMR sensors has been dramatically improved by more than 100 times in the past few years^11^. In a recent review which proposed a roadmap of magnetic sensor development^29^, the detectivity of TMR was expected to reach to 10 fT/√Hz in the future, corresponding to the level of SQUID. Ultimately, TMR will become so much more sensitive that the whole sensor package would be as small as 50 μm, the ideal MTJ size since no MFC would be necessary.

Tangential measurement is another advantage of TMR-MEG, which detects the largest signal component of MEG produced by the current below, and because the fixed sensing axis of the TMR sensor can detect neural current orientation below the sensor. Recently, some simulation studies have quantitatively examined the advantages of radial and tangential measurements. Normal or tangential measurement using on-scalp OPM arrays yielded 7.5 or 5.3 times higher signal power than SQUID magnetometers^30^, and a triaxial array of OPM offered dramatic improvement to differentiate real brain activity from sources of magnetic interference (external to the brain), as well as marked improvement in the elimination of artifacts caused by head movement^31^. At present, triaxial measurement is not available using a single TMR sensor. However, high density arrangement of small TMR sensors with orthogonal sensing axes will be able to clearly separate and localize multiple neural activities. For already known multiple neural activities, a limited number of TMR sensors can be placed on the scalp immediately above the sources with tangential sensing axis to the scalp and perpendicular sensing axis to each current orientation.

Currently, the sensitivity of TMR is inferior to that of OPM or SQUID. However, the development of much more sensitive TMR sensors has been indicated by our recent progress^11^ exceeding the roadmap^29^ 3 or 4 years ahead. Moreover, the several advantages of TMR described above will soon overcome at least some of the inferior sensitivity. First, the shorter distance from the source to the sensor, i.e., perfect attachment of the sensor to the scalp, will not improve both the signal sensitivity and the spatial resolution even without sophisticated algorithms for source estimation. Second, the wider dynamic range of TMR than OPM or SQUID will enable more flexible use of the MEG measurement, such as outside the shielded room. Third, the smaller size of future TMR sensors than OPM or SQUID will enable higher density spatial arrangement of multiple sensors. Fourth, the cheaper fabrication and running costs of TMR than OPM or SQUID will enhance the mass manufacture of MEG and other biomagnetic imaging systems.

The SEF amplitudes measured by TMR and SQUID sensors were comparable even though the SQUID was further away from the scalp in Fig. 3. We believe that the MFC of our TMR system was too large (26 mm) compared to the size of the signal source, so that the whole sensor system detected the averaged signal affected by lower SEF amplitudes at the slope rather than the highest peak. In addition, the combination of multiple channels is not feasible in the present TMR sensor due to relatively large size of the MFC and possible interactions within the field concentration. However, future improvement of the TMR sensitivity will enable smaller or no MFC, and thus three-dimensional multichannel MEG systems as well as measurement of larger SEF signals than possible by the conventional SQUID sensor.

In conclusion, our present success with scalp-attached and scalp-tangential MEG measurement will allow the development of non-invasive functional imaging of human organs with millimeter and millisecond resolutions.

## Methods

### Subject

A right-handed male subject, aged 27 years at the time of participation, was recruited from local residents through advertisements in a local town paper. The subject was not using any medications known to interfere with cognitive function, including benzodiazepines, antidepressants, or other central nervous system agents. He had no history of head trauma, mental disease, or diseases known to affect the central nervous system. This study was approved by the Ethics Committee of the Tohoku University Graduate School of Medicine for the SQUID-MEG part (#2021-21650) and Graduate School of Engineering for the TMR-MEG part (#2018-17A-16). Written informed consent was obtained from a subject. The study was conducted according to the principles expressed in the Declaration of Helsinki.

### SEFs using SQUID-MEG

Electrical stimulation was administered to the left median nerve of the subject. The electrical stimuli consisted of constant current biphasic pulses with duration of 0.3 ms delivered at 2.9 Hz. Stimulus intensity was set at 1.5 times the motor threshold to evoke a twitch of the thumb^32^.

SEFs were recorded in a magnetically shielded room constructed with 3 layers of high permeability nickel–iron alloy (mu-metal) and one layer of aluminum (Daido Plant Industries Co., Ltd.) using a 200-channel whole-head type axial gradiometer system (MEGvision PQA160C-RO; Ricoh, Tokyo, Japan).

The sensors are configured as first-order axial gradiometers with a baseline of 50 mm; each gradiometer coil is 15.5 mm in diameter. The sensors are arranged in a uniform array on a helmet-shaped surface at the bottom of a Dewar vessel, and the mean distance between the centers of two adjacent coils is 25 mm. Sensor field sensitivity (noise of the system) was 3 fT/Hz within the frequency range for SEFs^33,34^. The subject lay supine, with the head location determined by the positions of five fiduciary markers consisting of induction coils placed at known locations on the scalp. The head shape and coil positions were established using a three-dimensional digitizer (Fast SCAN Cobra; Polhemus, Inc., Colchester, VT) based on three-dimensional magnetic resonance (MR) images obtained for the subject using a 3 T MR system (Achieva; Philips Healthcare, Best, the Netherlands). The MEG signal was band-pass filtered between 0.03 and 2000 Hz and sampled at 10,000 Hz. To obtain the N20m response to median nerve stimuli, the data from 25 ms before to 175 ms after the stimulus onset were averaged 200 times. In subsequent off-line analysis, the averaged data were digitally band-pass filtered from 0.03 to 2000 Hz. The N20m response was identified visually as the first prominent peak at 20.2 ± 1.5 ms (mean ± standard deviation) after the onset^13^, with the isofield map showing anterior current orientation. The location of N20m source was estimated at the peak latency, using a model of equivalent current dipole with the best fit sphere for a subject’s head^35^. The source was superimposed on a three-dimensional MR image of the subject (Fig. 3d) using a MEG-MR image coordination integration system (MEG Laboratory; Ricoh), as well as used for simulation maps of both radial and tangential MEG measurement (Fig. 2).

### TMR-MEG system

Figure 1 shows a photograph and the schematic image of the developed TMR sensor with 74 MTJs, a facing pair of T-shaped MFCs, and the measurement circuit diagram used for the MEG measurement. Details of the TMR sensor are described elsewhere^11^. Device area of a MTJ was 50 × 50 μm^2^. Length of the series array of 74 MTJs was 6.7 mm. MTJ multilayer films were deposited on thermally oxidized Si wafers using an ultrahigh-vacuum (P_base_ < 1 × 10^−6^ Pa) sputtering system. The stacking structure was Sub. Si, SiO_2_/bottom electrode/Co_70.5_Fe_4.5_Si_15_B_10_ 140/Ru 0.4/Co_40_Fe_40_B_20_ 3/MgO/Co_40_Fe_40_B_20_ 3/Ru 0.9/Co_75_Fe_25_ 2/Ir_22_Mn_78_ 10/Ta 5/Pt 5/Ru 5 (in nm). The bottom CoFeSiB and CoFeB free layers show weak magnetic coupling, and the magnetization reversal process reflects that of the thick CoFeSiB layer with excellent soft magnetic properties^10,11,36^. For the preparation of very thin MgO barriers, the Mg layer of 0.7 nm was deposited on top of the bottom CoFeB layer and then oxidized by pure oxygen in the deposition chamber. To fully oxidize the Mg layer and obtain the intended thickness of the MgO barrier, the process of depositing Mg layers and in-situ natural oxidization was repeated several times sequentially^11,28^. The prepared MTJ multilayer films were microfabricated into MTJ arrays by photolithography and Ar ion milling. The TMR sensor has a structure in which 74 MTJs with device area of 50 × 50 μm^2^ are connected in series to reduce the 1/f noise^11,37–39^ and the length of the 74 MTJ array is 6.7 mm. After the microfabrication, the MTJ arrays were annealed using the double annealing process with a magnetic field of 0.3 T to realize linear output response against the external magnetic field^11,40^. The first and second annealing temperatures were 330 °C and 225 °C, respectively. The output of the TMR sensor predominantly changes with magnetic field applied in the short-side direction (sensing axis) of the MTJ array. On both sides of the MTJ arrays, a T-shaped MFC, which is effective for concentrating the external magnetic field^11,41,42^, was prepared as shown in Fig. 1. The MFCs of 300-nm thick Fe_73.5_Cu_1.0_Nb_3.0_Si_15.5_B_7.0_ films were deposited, and 0.5-mm thick Ni_80_Fe_20_ plates with T-shape were subsequently placed on the Fe_73.5_Cu_1.0_Nb_3.0_Si_15.5_B_7.0_ films. The T-shaped MFCs have a size of 12.5 mm in the direction of the sensing axis (vertical line of “T”) and 26.0 mm in the direction parallel to the MTJ array (horizontal line of “T”)^11^.

A TMR sensor was used as one of the resistors in the bridge circuit, and its output voltage was amplified and filtered. The bridge was supplied with ± 0.2 V, so the bias voltage of about 0.2 V was applied to the TMR sensor. The circuit to receive the signal from the bridge consisted of our homemade amplifiers and capacitor-resistor passive filters. The biomagnetic field signal measured by the TMR sensor was input to a two-stage amplification circuit with a total gain of 120 dB and passed through an analog bandpass filter from 0.8 Hz to 2 kHz. The signal output of the fabricated sensor system was 2.36 V/nT. The amplified and filtered signals were recorded on a PC at sampling rate of 2 kHz using an ADInstruments PowerLab 16/35 and LabChart8 software. The magnetic field signals were filtered in the software with a moving average (digital filter) from 20 to 200 Hz. The pulse signal of the stimulation was also measured using the analog-to-digital converter at the same time, and the MEG was integrated 9000 times by using the rising edge of the pulse as a trigger.

The sensitivity of our new TMR sensor was 940 fT/√Hz at 1 Hz, 200 fT/√Hz at 10 Hz, and 50 fT/√Hz at 1 kHz, which is about 100 times better compared to conventional magneto-resistive sensors^16^, and none of the previous magneto-impedance sensors^17^, fluxgate sensors^18^, or diamond quantum sensors^19^ have reached the high sensitivity level of our TMR sensors.

### Simulation of TMR and SQUID-MEGs

The TMR sensor used in this study has sensitivity in the horizontal direction of the scalp, which is different from the measurement direction of SQUID-MEG. Therefore, the tangential and radial magnetic flux densities generated by the N20m current were compared by simulations. The magnetic flux density on the scalp surface and SQUID measurement surface was calculated using a finite element method (Murata Software Femtet 2020.1.2.). The scalp model was an ellipsoid with radii of 10.3 cm in the front-back direction, 8.0 cm in the left–right direction, and 8.0 cm in the up-down direction, based on MR images and actual measurements, and the magnetic flux density was calculated when the measurement surface moved away from the scalp by 0.5 to 2.0 cm. The measurement surface of the SQUID-MEG was a sphere with a radius of 12.2 cm. The current of N20m in the brain was shaped as a thin cylinder with a length of 0.1 cm and a base area of 1 cm^2^ based on our previous study of SEFs for the median nerve and the individual hand digit stimulus^42^. The position and direction of the current were determined from the results of SQUID-MEG and MR imaging measurements. The magnitude of the current was adjusted so that the magnetic flux density in the radial direction matched the results of SQUID-MEG measurements. Here, the depth of the N20m current from the scalp was 2.4 cm at the shortest distance. The calculated magnetic flux density was plotted in two components: perpendicular to the N20m current on the scalp surface, and in the radial direction of the scalp. The plot perpendicular to the current on the scalp surface corresponds to the actual measurement direction of the TMR sensor, and the radial plot corresponds to the measurement direction of SQUID-MEG.

### Measurement of median nerve SEFs by TMR-MEG system

The subject underwent the same electrical stimulation in a magnetically shielded room as used in the previous SQUID-SEFs measurements. First, the subject wore a swimming cap to prevent any disruption caused by the hair, fixed with elastic tape. Second, the TMR sensor was placed at the current dipole position determined by the SQUID SEFs. Next, the TMR sensor was repositioned at 1-mm and 1-degree intervals to find the maximum amplitude. Finally, the TMR sensor was placed at a position perpendicular to the active current direction to determine the maximum horizontal sensitivity to the scalp.

## Data Availability

The data that support the findings in this study are available from the corresponding author on reasonable request.
